# Essays on Infant Therapeutics

**Published:** 1855-02

**Authors:** 


ART. VIII.—*Essays on Infant Therapeutics: to which are added, Observations on Ergot; History of the Origin of the Use of Mercury in Inflammatory Complaints; together with the Statistics of the Deaths from Poisoning in New York in the years* 1841–2–3. *Second Edition.* By JOHN B. BECK, M. D., Prof. Mat. Med., etc. New York: S. S. and W. Wood. 1855.

This little 12mo. has been so long before the public that it is unnecessary for us to afford it an extended notice. It is characterized by an intelligent moderation, and small as it is in size, there is little doubt that it has exerted as powerful an influence upon therapeutics as almost any work of its day. We have heard its precepts cited as authority at consultations in a log shanty, and have known it to modify the treatment of infantile disease very largely.

It is certainly a very valuable work, and should go as the companion and antidote of most of our work on the diseases of infants, to modify their reckless recommendation of the use of the most powerful drugs on the susceptible constitutions of children.

For sale by PHINNEY & Co. H.

